# An Empirical Study Analyzing the Moderating Effect of Supervisor Support and Mediating Effect of Presenteeism among Eustress, Distress, and Innovative Behavior

**DOI:** 10.3390/bs13030219

**Published:** 2023-03-02

**Authors:** Amna Anjum, Yan Zhao, Naeem Faraz

**Affiliations:** 1 School of Management, Shanghai University, Shanghai 200444, China; 2 International Cultural Exchange School (ICES), Donghua University, West Yan’an Road 1882, Shanghai 200051, China

**Keywords:** distress, eustress, presenteeism, supervisor support, innovative work behavior

## Abstract

Purpose: This article aims to illustrate that stress is not always a negative experience as it can have both positive and negative outcomes. The term “eustress” describes positive stress, while the term “distress” describes negative stress. To date, research on eustress is in the infancy stage. There are approximately 306 items that can be found in the Web of Science core collection for “eustress”, while there are 184,714 items found for “distress”. Few studies have examined the relationship between presenteeism, stress, and innovative behavior. Thus, the mechanism underlying this pathway still needs to be fully understood. Materials and Methods: A survey was conducted among 350 medical healthcare professionals from Pakistan. With the help of SPSS and AMOS, the data were analyzed and the combined effects of the variables were also investigated. Results: According to the current study, a mediation effect has been observed between innovative behavior and stress (eustress and distress). However, supervisor support moderates the relationship between stress and presenteeism and, likewise, between presenteeism and innovative behavior. Conclusion: Our analysis of variables establishes empirically robust relationships between the innovative behavior of medical healthcare professionals and the two different dimensions of stress. In addition, it describes a hypothetical alternative situation that explains how employees’ innovative work behavior is affected by eustress and distress in the presence of supervisor support. This study could have implications for improving medical healthcare professionals’ ability to incorporate innovative behavior into their practice in an effective manner in the future.

## 1. Introduction

In this modern era, innovation has become a necessity rather than a choice for most organizations. The ability of the employees to plan and execute new ideas is an essential characteristic of the administration that wants to succeed in this age of technology, especially during the COVID-19 pandemic situation. According to researchers, innovative work behavior is finding and applying new creative ideas to obtain organizational performance by securing the resources to develop actionable plans. A person or organization that practices IB introduces and applies new ideas to improve performance. Many studies claim that innovation in any organization comes through the innovative behavior (IB) of the employees, which is needed for any organization, either non-profit or profit. Innovative behavior is important for employees because it drives creativity and continuous improvement in the workplace. Employees who engage in IB are more likely to find better ways of doing things, which can lead to increased productivity and efficiency. When employees are encouraged by their supervisors to be innovative, they are more likely to come up with the new idea that can streamline processes and make the work easier. IB allows employees to feel a sense of ownership and pride in their work. When employees are encouraged to contribute their ideas and suggestions, they feel valued and respected, which enhances their job satisfaction. This leads to the development of new ideas, products, and services that can benefit the organization and its customers. Organizations that foster innovative behavior among their employees are more likely to stay ahead of the competition. Innovation helps to keep businesses relevant by adapting to changing business conditions, anticipating customers’ needs, and developing new products and services.

In the health sector where innovation is no longer an option, especially during COVID-19, organizations focus on innovation to cope with pandemic consequences. Coronavirus disease (COVID-19) has been causing outbreaks of infectious diseases worldwide since the end of 2019. Global health authorities stated that COVID-19 is an emergency and a global health concern in January 2020. Pathogens are emerging and re-emerging globally [1]. In unrelenting efforts to contain the epidemic, Medical Healthcare Professionals (MHCPs) have played a key role in reducing the effects of COVID-19. Several surveys indicate that employees worldwide are affected by the pandemic [2], including Pakistan. MHCPs’ IB compromises stress, long working hours, irregular shifts, and increasing demands caused by the COVID-19 pandemic affecting the employment situation. Not only have these problems negatively impacted the MHCPs, but they have also diminished enthusiasm and initiative ability, which in turn creates hurdles in the innovative behavior of MHCPs.

To address the emergency of COVID-19 all over the world, researchers recently shifted their focus to evaluating employees’ IB [3]. In recent years, many articles have been written to see innovation in the workplace, focusing mainly on different stressors, which cause hurdles in the IB of employees. Studies have shown that more stressors occur during the pandemic situation and that the heavy workload forces employees to remain in the workplace, which increases their office time and attendance, ultimately promoting the presenteeism of MHCPs [4,5]. It is well-documented that presenteeism is costlier than absenteeism. Annually, presenteeism costs $36 billion in indirect costs, with a 13.2% productivity loss [6,7]. The study on Chinese chief nurses estimated that presenteeism results in an average decline of 21.01% in productivity [8,9]. During the epidemic, presenteeism became more prevalent. This pandemic also caused more challenges for healthcare workers. Despite saving others’ lives, a higher presenteeism rate was observed in MHCPs during the pandemic. MHCPs were facing increased work pressure as a result of the pandemic, resulting in a continual increase in presenteeism, consequently resulting in a decrease in innovative behavior.

The relationship among stress, presenteeism, and innovative behavior has received significant attention from researchers. However, some research gaps in these areas still need to be addressed. Although, the relationship between stress and presenteeism is well established, more research is needed to understand the mechanism behind this relationship. The effects of distress and eustress on presenteeism need further investigation with the combination of other variables, which have not been studied yet. Additionally, research is needed to determine the most effective interventions for reducing stress and presenteeism in the workplace. To cope with this issue, we have investigated the role of supervisor support as a moderator. Furthermore, there is a need for more research on the factors that influence innovative behavior, including the role of individual and organizational characteristics and the impact of stress and presenteeism on innovative behavior. Additionally, there is a need for more research on the impact of innovative behavior on organizational outcomes, such as productivity and profitability. 

Considering the above discussion, we selected two main variables related to IB based on literature and epidemiological background: work stress and presenteeism. Supervisor support was examined as a moderator to investigate how much it can compensate for the effects of work stress on IB among MHCPs.

This paper is organized in the following manner. A detailed description of the variables that have been evaluated is provided in Section 2, along with a literature review focusing on eustress, distress, presenteeism, supervisor support, and innovative behavior. The aim is to identify and measure each of the constructs of the research model being assessed. There is also a research model presented in this section that claims that a direct relationship exists between the different dimensions of stress (eustress and distress) and the innovative behavior of individuals. In Section 3 and Section 4, the methods used in this study are explained in detail. The results derived from SPSS and AMOS are presented in Section 5 of the paper. Finally, the last part of the article focuses on the empirical results and their implications for management practitioners.

## 2. Literature and Hypotheses Development

### 2.1. Eustress and Distress

According to the Job Demand-Resource Model, stress at work results from an imbalance between job demands and resources. When demands are high, and resources are low and employees are more likely to experience stress [10]. Sullivan [11] points out that stress is never always a negative experience as it can be both positive and negative. Working in a stressed atmosphere could cause both negative and positive stress in an individual. Stress has been associated with several factors, which are discussed by Amna et al. [12,13]. Distress is a feeling of being threatened by demands that are perceived as overwhelming. The feeling of powerlessness is familiar among those experiencing distress because they feel they have no control over their jobs or lives. Individuals’ psychological and physical situations are strongly associated with their working attitudes [14,15,16,17]. Distress at work can hurt the psychological situation of employees and their ability to perform innovative tasks. Whereas eustress is usually viewed as tolerable stress and is typically seen as a good stress factor. It is viewed as a means of upholding one’s progress and achieving one’s goals, such as a controllable assignment that requires minimal effort. It is believed that the eustress effect leads to an increase in positive emotions, performance, motivation, and innovative attitudes, which leads to an increase in IB as well. Since, the eustress effect incites positive emotions, increases performance, and motivates individuals, employees and supervisors need to develop the environment of eustress and support innovative ways of working to better respond to the demands in a determinative context. By providing opportunities for personal improvement or growth, controllable stressors, such as heavy workloads and time pressures, tend to develop an environment of eustress and promote IB among employees. However, negative stressors, such as job insecurity caused by COVID-19, hinder personal development and achievement and suppress IB in the workplace.

**Hypothesis** **1.** *Eustress at work positively impacts innovative behavior*.

**Hypothesis** **2.** *Distress and innovative behavior have a significant negative relationship*.

### 2.2. Presenteeism

Presenteeism refers to employees attending work despite being physically or mentally unwell. According to many scholars, presenteeism occurs when employees show up for work but do not meet their full potential due to stress or if they go to work when they do not feel well. This can result in decreased productivity, decreased work quality, and a lack of engagement. The theory of presenteeism is based on the notion that employees feel pressure to attend work regardless of their health. This pressure may come from a variety of sources, including a strong work ethic, a culture that values face time, or an employee’s fear of job loss. Several theories attempt to explain why presenteeism occurs, including Role theory [18] and Social norms theory [19]. Role theory suggests that employees feel a sense of duty and responsibility to fulfil their roles and perform their job duties, even when they are unwell. Social norms theory states that employees attend work because they believe it is the form of expected behavior from them and they also have a fear of being perceived as not committed to the job if they take time off. 

In comparison to absenteeism, presenteeism causes a more severe loss than absenteeism. Many reports have been published that indicate that organizations suffer more losses from presenteeism than from absenteeism [20,21]. There are several studies analyzing the monetary loss due to absenteeism and presenteeism. Some surprising results came from the studies that there is a five times greater monetary loss associated with presenteeism than absenteeism, with a ratio of 1:5 [22]. As a result, the report clearly shows that presenteeism is one of the greatest threats to an organization as it can cause a reduction in innovative behavior and downgrade the quality of services in the workplace. A second report indicates that the indirect costs of presenteeism have reached a national average of $36 billion, with a mean productivity loss of 13.2% due to presenteeism [23]. It is also estimated in China that the average presenteeism among the chief nurses’ subordinates reduces the nurses’ productivity by 21.01% each year [24,25,26,27]. A study has also shown that, in addition to the productivity loss that occurs as a result of presenteeism, presenteeism also leads to an 18% increase in the number of accidents and medical errors that occur among patients due to presenteeism [28,29,30]. As a result of the unique characteristics of MHCPs, such as shift work, inflexible working schedules, extended working hours, and heavy workloads, MHCPs experience an extremely high level of stress, which decreases their IB and makes it almost impossible for them to perform their job effectively in the workplace.

**Hypothesis** **3.** *Innovative behavior is negatively correlated with presenteeism.*

**Hypothesis** **4.** *Presenteeism mediates between distress and innovative behavior.*

**Hypothesis** **5.** *Presenteeism mediates between eustress and innovative behavior.*

### 2.3. Supervisor Support

**Figure 1:**
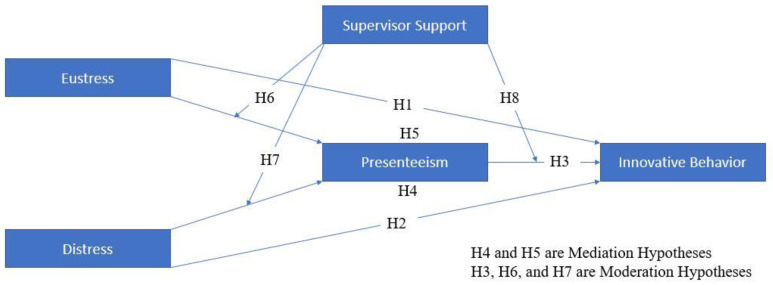
Proposed model of the study.

Supervisory support theory [31] refers to the idea that supervisors play a crucial role in shaping the experiences and outcomes of employees in organizations. According to this theory, employees are more likely to be satisfied, committed, and productive if they perceive their supervisors as supportive, understanding, and responsive to their needs and concerns. Supervisor support can take many forms, including emotional, informational, and instrumental support. The theory suggests that a supportive supervisory relationship can enhance job satisfaction, reduce stress and burnout, and improve organizational outcomes, such as employee turnover, absenteeism, and presenteeism. Additionally, it has been shown that when employees receive support from their supervisors, they are more likely to engage in behaviors that support the organization, such as helping others, going above and beyond their job duties, and providing suggestions for improvement and innovation. The support employees receive from supervisors [32,33,34,35] plays a key role in organizational success. Supervisors try to develop social bonds among their co-workers to develop their ability to collaborate and respond to one another to ensure that their organization succeeds in the long term. Support from supervisors is a measure of the degree to which employees distinguish their administrators as being willing to support them regarding work-related problems or fulfilling their assigned tasks or targets. It can be measured by how often workers observe their managers being available to help them. Providing supervisors with the support they need employees can segment their information and knowledge with their subordinates. A detailed representation of the model can be seen in Figure 1.

**Hypothesis** **6.** *Supervisor support moderates the relationship between eustress and presenteeism.*

**Hypothesis** **7.** *Supervisor support moderates the relationship between distress and presenteeism.*

**Hypothesis** **8.** *Supervisor support moderates the relationship between presenteeism and innovative behavior.*

## 3. Measures

All items were administered using only English scales for the measure. The English scales were verified using Cronbach’s alpha value—a reliability measure. Considering the work of Cavanaugh et al. [36], we divided both eustress and distress into two categories. Eustress and distress were measured with the 11 items. Five items were related to eustress, and six were related to distress. The Eustress-Distress Scale has a five-point Likert scale (1 = no stress; 5 = great stress; Cronbach α=0.928 and 0.830).

Stanford Presenteeism Scale (SPS-6) was used to measure presenteeism in our study. The SPS-6 is a well-known and quite popular instrument among researchers for measuring presenteeism. It has good psychometric properties and comprises six items. To ensure that SPS-6 scores reflect the magnitude of presenteeism, we reverse the scores for the presenteeism scale and then sum up the scores. The values were reversed to their mirror image (1 = 5, 2 = 4, 3 = 3, 4 = 2, and 5 = 1). Hence, more presenteeism has been mentioned by large values. To determine the innovative attitude of employees, Albort-Morant developed a questionnaire that consisted of 21 items with a Cronbach’s coefficient of 0.91 [37]. An employee’s innovative work behavior can be classified into three categories depending on how they operate: the innovation system (10 items), the competitors and technology (7 items), and new services (4 items). Based on eight items [34], Eisenberger et al. developed a survey of supervisor support. Three are about whether my supervisor will help me, two are about how valuable my suggestions are, and two are about how concerned he is about my health. A Likert scale is used to categorize the responses with 1 emphasizing strong disagreement and 5 emphasizing strong agreement for all the studied variables. Please refer to the Supplementary Material for more about the questionnaires.

## 4. Procedure

Following ethics approval from hospital ethics committees, medical healthcare practitioners from selected public and private hospitals in Pakistan were surveyed. Before participating in the study, all subjects provided informed consent. The Punjab Institute of Mental Health (PIMH) Ethics Committee approved the protocol by the Declaration of Helsinki. The Institutional Review Board approved the research on 2 July 2022. Participants’ voluntary and confidential consent was obtained. They were completely free to decline participation in this study, and the doctors were assured that their refusal would not affect their work or personal lives. Data integrity was ensured by keeping all the data secure and confidential and allowing only the research team to access them. A simple random sample of 350 employees working at different levels of responsibility in hospitals of Lahore (Pakistan) were surveyed. We explained to the participants what the study was all about and how confidentiality would be protected. In addition, they were also given a brief survey that included multiple items, including demographic data, that they needed to fill out. Employees whom the hospital employed on an official basis were eligible to participate in the survey. Upon confirmation that they were eligible for the study, they were then asked to complete a composite survey.

## 5. Statistical Analysis

As part of the statistical analysis, IBM SPSS version 24 and IBM AMOS version 24 were used for all the calculations. Statistical characteristics of our sample were measured before hypothesis testing. A structural equation model was used to test the mediating and moderating effects.

### 5.1. Descriptive Statistics

All the descriptive analyses and the summary of the collected data have been presented in Table 1.

### 5.2. Results

From Table 2, age significantly positively relates to position, experience, distress, presenteeism, innovation, and supervisor support, whereas age has a significant negative relation with eustress. When age increases, stress level drops. Eustress needs vitality, which goes down with age. Similarly, positions have a significant positive relationship with experience, distress, innovative work behavior, and supervisor support. The position has a negative relation to presenteeism. As it is said, “great responsibilities come with great positions”, so the level of presenteeism will go down with a high position. The position has an insignificant relationship with eustress. Also, experience has a significant positive relationship with eustress, innovation, and supervisor support but has a significant negative relationship with distress and presenteeism.

Furthermore, we can see a significant positive relationship between eustress and innovative behavior, which supports our H1 and it is consistent with the study of Pie Hu Xie [38]. On the other hand, distress has significant negative relation with innovative behavior, which supports our H2. This result also matches with the findings of Pie Hu Xie [38]. Presenteeism is significantly negatively correlated with innovative behavior, which supports our H3.

### 5.3. Results of Exploratory Factor Analysis

To categorize the data set (see Table 3), exploratory factor analysis (EFA) was used to analyze all of the items based on factor analysis. To assess sampling adequacy, Cerny, C.A., and Kaiser incorporated the Kaiser–Meyer–Olkin (KMO) test [39] as well as Bartlett’s sphericity test [40]. At the level of *p* 0.000, the values were calculated to be 0.833 and 4232.555, respectively. A KMO of 0.6 as a minimum value [41] has been suggested by researchers who have ranged KMO values from 0 to 1.

Initially, twenty-one items were in the IB questionnaire. However, on performing the EFA, we figured out that items from 18 to 21 contributed little, so with the help of EFA, the items were reduced to 17. Similarly, for supervisor support, there were eight items in total. However, item number seven was deleted after the implementation of EFA.

### 5.4. Confirmatory Factor Analysis

**Figure 2:**
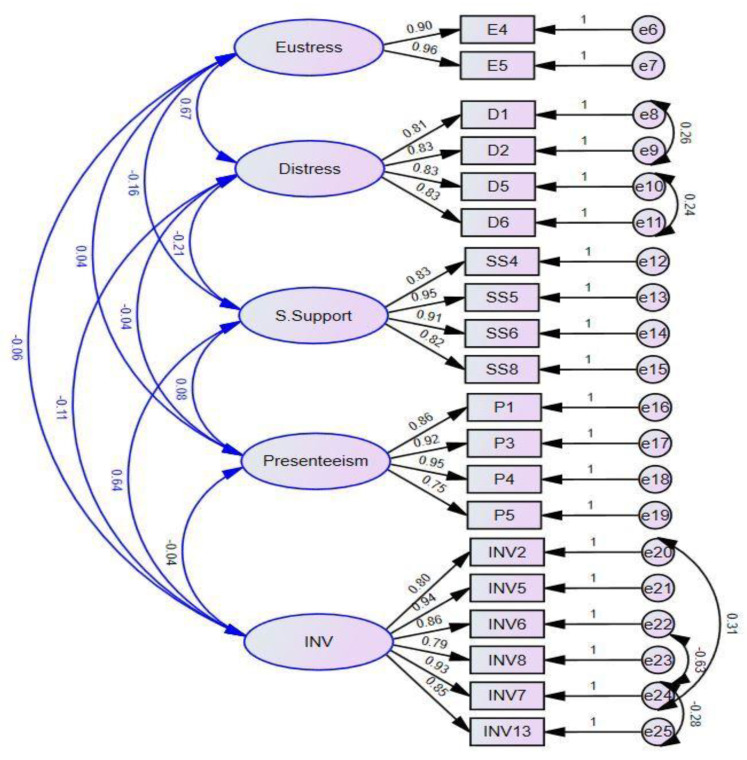
Confirmatory factor analysis.

AMOS 24 has been used to analyze each variable’s convergence and discriminant validity. Since all pre-developed questionnaires have been modified to maintain the convergent validity of all variables, maintaining the convergent validity has become reasonably necessary. There was a significant difference between the average variance extracted (AVE) and the average factor loadings, which were found to be greater than 0.65 and 0.7 respectively. Hence, our five-factor CFA meets the criteria of convergent validity by the standards outlined in Figure 2.

In line with the literature, the average variance of all variables (AVE) should be significantly higher than the maximum/average shared square variances (MSV and ASV), which can be seen in our case as well (see Table 4). It was found that there was less than a square root correlation between each construct and the AVE of its square root. In Table 5, the global fitness of the four variables is presented to show the model’s goodness of fit.

### 5.5. Mediation Analysis

The direct and indirect relationships between the variables have been observed using the structural equation modeling (SEM) technique and are presented in Table 5 of the results.

**Figure 3:**
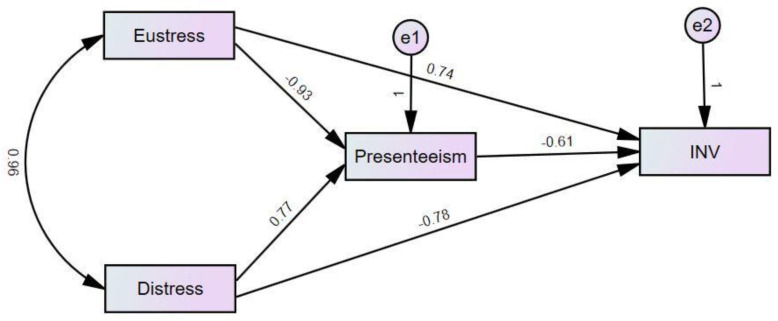
SEM analysis proposed model.

Figure 3 is part of Figure 1. In Figure 1, we have incorporated both mediation and moderation variables. However, Figure 3 focuses on the SEM analysis of the mediating variable. Furthermore, in Table 6, we can see that presenteeism has a significant positive relation to distress and a significant negative relation to innovative behavior. However, distress has a significant negative relation with innovative behavior. Furthermore, the direct effect is less than the indirect effect. This suggests that presenteeism partially mediates between distress and innovative behavior, which supports our H4. Similarly, eustress has a significant negative relation with presenteeism and a significant positive relation with innovative behavior; however, the direct effect is less than the indirect effect, so according to Barron and Kenny, there is a partial mediation of presenteeism between eustress and innovative behavior, which supports our H5.

### 5.6. Moderated Mediation Analysis

As shown in Table 7, the direct and indirect effects of moderation have been observed through the structural equation modeling technique (SEM).

There is a significant positive relation between eustress and presenteeism and a significant negative relation between supervisor support and presenteeism. However, eustress and supervisor support have no significant relation to presenteeism. Hence, supervisor support does not moderate between eustress and presenteeism, which negates our H6. However, distress has a significant relation with presenteeism, and the interaction term of distress and supervisor support also have significant relation, so supervisor support moderates the relationship between distress and presenteeism; hence our H7 is supported.

### 5.7. Mediated Moderation Analysis

A moderated mediation was conducted between presenteeism and innovative behavior, and the changes were analyzed.

Table 8 shows that presenteeism is significantly related to IB, and supervisor support is significantly related to IB. There is also a significant relationship between interactive variables and IB. In other words, the supervisor supports moderate presenteeism and innovative behavior, which supports H8.

## 6. Discussion

More attention should be paid to eustress in the literature due to its importance for innovative performance and competitiveness. The results of our study shed light on the influencing factors, moderators, and boundaries of employee innovation. We predicted that stress increased presenteeism indirectly, which negatively affected IB, whereas presenteeism and supervisor support played mediating and moderating roles in this association. In Pakistan, 350 medical healthcare practitioners from different hospitals participated in an investigation. It was observed that individuals’ IB was positively affected by eustress. These findings showed how eustress can motivate people to perform at their peak, take on challenges with enthusiasm, and seek opportunities for learning, growing, and enhancing expertise. This will make it possible for employees to exhibit IB. However, distress negatively influenced IB in our study. These findings are consistent with Pie Hu Xia [38]. Pie Hu Xie’s finding indicates that eustress influences employees’ innovative behavior positively, whereas distress reveals negative effects on employees’ innovative behavior.

A significant negative relationship was found between eustress and presenteeism. However, this result was different from Huirui Jian’s [42]. According to Huirui Jian [42], eustress was positively related to presenteeism, but in our study eustress was negatively associated with presenteeism. As is evident from other studies [11,43,44], there is a very slight difference between eustress and distress; when the stress level reaches the point of no return, it converts into distress, and employees’ performance becomes compromised. On the other hand, as long as a person is energetic and takes work pressure or load as an opportunity to learn, it helps them deal with the workload and pressure and minimizes presenteeism.

Looking at this fact in the context of Pakistani society, it is true, according to the current economic situation of Pakistan workers, especially in the health sector. It is challenging for workers to support their families due to the high inflation rate. Therefore, no one takes the risk of changing jobs or leaving a job. To give their best, everyone tries their best to continue their job and meet their job requirements. Also, unemployment occurs in almost all sectors, especially in the health sector. To sustain themselves in their jobs, employees try to bear as much stress as possible. Our study showed that the decreased presenteeism level influenced innovative work behavior positively. A significant negative relationship has been observed between presenteeism and IB, but the beta value of the direct relation is lower than the indirect values, which indicates that there is partial mediation of presenteeism between eustress and innovation. This shows that employees are trying to accommodate as much stress as they can, which is categorized as eustress, but when eustress reaches its breakeven point, this eustress converts into distress, and this shows its severe impact on the employee’s performance. Since 2020, due to the pandemic, healthcare professionals are under lots of stress, especially due to workload and overtime work, which impacts the performance of the MHCPs.

Furthermore, supervisor support does not moderate between presenteeism and eustress. Eustress has a significant negative relation with presenteeism, which means if there is more eustress, it decreases the presenteeism rate. In this case, there would be no need for any moderator, such as supervisor support. Employees may have a high tolerance level and focus on their work if they are pleased, interested, and prepared to face encounters and they consider them a part of training that could help them grow professionally.

Similarly, there is a significant relationship between distress and presenteeism as well as the interaction term (distress*supervisor support). Hence supervisor support moderates distress and presenteeism. Every human has a certain level of tolerance. It takes a second to fall into distress. MHCPs in Pakistan are also under great stress due to the pandemic. Supervisor support played a significant role in compensating for and reducing job threats and workload to enhance their performance. According to Geme Albort-Morant [45], work-related stress leads to negative outcomes, and supervisor support does not make a significant impact to improve the performance of the employee. However, in our studies supervisor support plays a significant role to enhance the innovative behavior of the employee. 

The supervisor can take several approaches to prevent job stress in an organization. The first step is to identify potential sources of stress within the organization and to deal with these issues as soon as possible. Employees’ mental and physical health can be improved by supervisors who can minimize job-related stress. Several primary interventions could be used to reduce job stress among employees, including providing respite time, allowing them to nap when necessary, and encouraging them to participate fully in decision-making and planning to make them feel as if they are essential to the organization as a whole. It may be necessary to increase the time and resources available for supervisors to enhance their innovative work behavior and increase the time and resources available for specific job tasks. Supervisors should match employee job descriptions with their skills and qualifications to improve IB. To motivate their employees, supervisors should amend their policies to provide clear paths to promotions and rewards. Despite ill health, employees can eventually develop IB by working hard and striving for rewards and promotions.

Furthermore, we can see from Table 6 that presenteeism is inversely proportional to IB, which is understandable. When there is a high presenteeism rate, the performance of the people will go down, ultimately affecting their innovative behavior. Here, supervisor support played a vital role in enhancing the IB of an employee, as we can see from the table that presenteeism and interaction terms of presenteeism and supervisor support have significant relation with IB. A supervisor is the ultimate authority in any organization who has direct interaction with the employee and can better understand the problems of their subordinates. Also, subordinates are comfortable sharing their problems with their direct boss rather than with high authorities. 

## 7. Theoretical Contributions

Several contributions are made to the literature. The findings of our study extend our understanding of how job stress inhibits innovation at work. Research has shown that job stress can affect involuntary work behaviors, but the mechanisms for these effects are largely unknown. To ensure that employees can function effectively in uncertainty, it is essential to take a ‘process lens’ approach. In this study, we investigate the mediating and moderating roles of presenteeism and supervisor support among MHCPs to understand how job stress affects innovative work behavior. Hence, we contribute to understanding how stressful job conditions affect innovation.

The study contributes a crucial insight by identifying supervisor support as a critical buffer against negative job stress and helping employees remain psychologically intense. Support from supervisors can help workers retain a passion for their jobs when they feel insecure. This allows them to maintain motivation and, consequently, implement innovative strategies. Employee intrinsic motivation and IB have tended to be the focus of current working conditions.

Study results show that supervisor support can reduce employee exposure to adverse workplace conditions, including job insecurity and high workload, especially during the COVID-19 pandemic. MHCPs in Pakistan are not well studied in terms of supervisor support’s impact on creativity and IB. Research has shown that supervisor support can also promote innovation in addition to improving health.

### Implications for Practice

According to Cavanaugh et al. [36], the current study’s findings on stress have practical implications. Hospitals and healthcare environments can improve employee motivation and happiness through perfect supervisor intervention while reducing job stress. Organizational policies should be adjusted to accommodate this trend by clearly stating what is expected of employees and allowing them to advance. It is observed that possible benefits from stress are met when a minimum level of stress is maintained. Destructive effects of distress were observed when the stress is beyond the employee’s control. Maintaining a minimum level of distress should be the goal of every organization.

## 8. Conclusions

In this study, we discussed the relationships among eustress, distress, presenteeism, supervisor support, and innovative behavior. The influence of stress to organizational members’ innovative behavior was successfully analyzed, whereas the mediation effect of presenteeism and the moderation effect of supervisor support were also identified. We found that eustress motivates personnel to fully develop innovative behavior, but distress demotivates personnel to fully develop innovative behavior. However, this demotivation can be overcome with suitable supervisor support. It is also observed that presenteeism mediates between eustress, distress, and innovative behavior. However, we can minimize this mediation by introducing suitable support from supervisors. Supervisor support plays a vital role in developing innovative behavior among employees. Future research should analyze relevant concepts using high working pressure and key predictive factors or moderators of employees’ innovative behavior. We think that this will enrich the exploration of organizational employees’ sustained innovative behavior and contribute to the advancement of theory and application to practice.

### Limitations and Future Research

The study has some limitations, for example, everything in the study is self-reported. Intentionally or unintentionally, people may not report themselves accurately. In the absence of an experimental design, causal conclusions cannot be. There may be another variable that may be problematic and was overlooked throughout the study. There may be an unanticipated relationship due to a third variable.

Furthermore, the findings of this study cannot be generalized since the sample consisted solely of medical healthcare workers. They are unique to Pakistan, mainly due to their work environment or cultural context, so the results cannot be applied to other occupations based on the current sample.

Since Pakistan is a big country, it is tough to extrapolate the results to the entire country. 

We could only collect data from Lahore because it was challenging to contact medical healthcare practitioners during this unprecedented pandemic situation. We had to take strict precautionary measures to visit the hospital and collect the data. We urge that the study be carried out over a more extended period and that the results be verified in a broader range of cities in Pakistan.

## Figures and Tables

**Table 1:** Descriptive statistics of the demographic variables.

Gender	Man	105 (30%)
	Woman	245 (70%)
Age (years)	<30	35 (10%)
	30–39	175 (50%)
	40–49	105 (30%)
	50–59	24 (7%)
	≥60	11 (3%)
Marital Status	Married	298 (85%)
	Single	49 (14%)
	Other	3 (1%)
Position	Consultant	35 (10%)
	Demonstrator	81 (23%)
	Registrar	109 (31%)
	Medical Officer	70 (20%)
	Postgraduate trainee	56 (16%)
Experience	0–3	42 (12%)
(years)	4–6	84 (24%)
	7–9	122 (35%)
	10–12	70 (20%)
	More than 12	32 (9%)

**Table 2:** Correlations among the studied variables.

Variable	Age	Position	Experience	Eustress	Distress	Presenteeism	Innovation	Supervisor Support
Age	1	-	-	-	-		-	
Position	0.40 **	1	-	-	-		-	
Experience	0.45 **	0.35 **	1	-	-		-	
Eustress	−0.20 **	0.25	0.32 **	1	-		-	
Distress	0.40 *	0.25 **	−0.4 **	0.96 **	1		-	
Presenteeism	0.40 **	−0.25 *	−0.32 **	−0.194 *	0.30 *	1	-	
Innovation	0.32 **	0.20 **	0.41 **	0.123 **	−0.19 **	−0.564 *	1	
Supervisor Support	0.16 **	0.30 **	0.12	−0.263 **	0.23 **	0.242 *	0.702 **	1

* Significant at 0.01 < *p* < 0.05; ** Significant at 0.001 < *p* < 0.01.

**Table 3:** Factor loadings, pattern matrix, and communalities.

Items	Factor’s Loadings						Communalities
Eustress1	0.75	0.840					0.735
Eustress2	0.73	0.814					0.746
Eustress3	0.77	0.776					0.723
Eustress4	0.90	0.726					0.743
Eustress5	0.96	0.732					0.772
Distress1	0.81		0.820				0.794
Distress2	0.83		0.801				0.723
Distress3	0.75		0.549				0.708
Distress4	0.73		0.651				0.718
Distress5	0.83		0.824				0.805
Distress6	0.83		0.918				0.771
Presenteeism1	0.86			0.837			0.696
Presenteeism 2	0.70			0.578			0.713
Presenteeism 3	0.92			0.956			0.876
Presenteeism 4	0.95			0.874			0.857
Presenteeism 5	0.75			0.862			0.825
Presenteeism 6	0.75			0.794			0.798
Supp. Supot.1	0.73				0.628		0.652
Supp. Supot.2	0.75				0.816		0.821
Supp. Supot.3	0.71				0.819		0.791
Supp. Supot.4	0.83				0.827		0.791
Supp. Supot.5	0.95				0.873		0.877
Supp. Supot.6	0.91				0.777		0.818
Supp. Supot.8	0.82				0.738		0.791
INV1	0.73					0.785	0.572
INV2	0.80					0.643	0.732
INV.3	0.77					0.881	0.763
INV.4	0.73					0.689	0.776
INV.5	0.94					0.705	0.808
INV.6	0.86					0.829	0.808
INV.7	0.93					0.863	0.778
INV.8	0.79					0.867	0.732
INV.9	0.71					0.891	0.632
INV.10	0.73					0.664	0.799
INV.11	0.72					0.741	0.693
INV.12	0.71					0.729	0.650
INV.13	0.85					0.704	0.747
INV.14	0.76					0.811	0.758
INV.15	0.77					0.843	0.650
INV.16	0.76					0.851	0.828
INV.17	0.72					0.916	0.733

**Table 4:** Average variance extracted, composite reliability, and collective Cronbach alpha.

Variables	Average Variance Extracted	Composite Reliability	Cronbach Alpha
Eustress	0.66	0.70	0.89
Distress	0.68	0.71	0.91
Presenteeism	0.75	0.76	0.85
Innovative Behavior	0.69	0.77	0.92
Supervisor Support	0.73	0.78	0.94

**Table 5:** Model fitness.

	Direct Effect	Indirect Effect
GFI	0.824	0.912
AGFI	0.834	0.893
TLI	0.937	0.892
CFI	0.949	0.832
RMSEA	0.079	0.055

**Table 6:** Hypotheses testing.

Hypothesis Tested	Relations	β Coefficients	*p*-Value	Remarks
H1	Eustress→Innovative Behavior	0.74	0.004	Accepted
H2	Distress→Innovative Behavior	−0.78	0.009	Accepted
H3	Presenteeism→Innovative Behavior	−0.61	0.000	Accepted
H4	Distress→Presenteeism→Innovative Behavior	0.77	***	Accepted
H5	Eustress→Presenteeism→Innovative Behavior	−0.93	***	Accepted

*** Significant at 0.0001 < *p* < 0.001.

**Table 7:** Direct and indirect relations of study variables.

Hypothesis Tested	Relations	Beta Coefficients	*p*-Value	Remarks
H6	Eustress×Supervisor Support→Presenteeism	0.64	0.094	Not Accepted
H7	Distress×Supervisor Support→Presenteeism	−0.45	0.004	Accepted

**Table 8:** Moderated behavior of supervisor support between health and innovative work behavior.

Hypothesis Tested	Relations	Beta Coefficients	*p*-Value	Remarks
H8	Presenteeism * Supervisor Support→Innovative Behavior	0.65	***	Accepted

*** Significant at 0.0001 < *p* < 0.001.

## Data Availability

The data presented in this study are available on request from the corresponding author.

## Supplementary Materials

The following supporting information can be downloaded at: https://www.mdpi.com/article/10.3390/bs13030219/s1, Questionnaire about Eustress and Distress; Questionnaire about Presenteeism; Questionnaire related to innovative work behavior.
