# Supplementary material for: An Empirical Study Analyzing the Moderating Effect of Supervisor Support and Mediating Effect of Presenteeism among Eustress, Distress, and Innovative Behavior
Source: Behav Sci (Basel). 2023 Mar 2;13(3):219. doi: 10.3390/bs13030219 (PMC10045315; doi:10.3390/bs13030219)
Supplement: Supplementary file 1 [file behavsci-13-00219-s001.zip › behavsci-2159556-supplementary.pdf]

The survey was conducted online and in person in July 2022.

Questionnaire about Eustress and Distress

1	E1: The number of projects and or assignments I have.	1	2	3	4	5
2	E2: The amount of time I spend at work.	1	2	3	4	5
3	D1: The lack of job security I have.	1	2	3	4	5
4	D2: The volume of work that must be accomplished in the allotted time.	1	2	3	4	5
5	E3: The degree to which politics rather than performance affects organizational decisions.	1	2	3	4	5
6	D3: The inability to clearly understand what is expected of me on the job.	1	2	3	4	5
7	D4: The amount of red tape I need to go through to get my job done.	1	2	3	4	5
8	D5: Time pressures I experience.	1	2	3	4	5
9	E4: The scope of responsibility my position entails.	1	2	3	4	5
10	E5: The amount of responsibility I have.	1	2	3	4	5
11	D6: The degree to which my career seems "stalled"().	1	2	3	4	5

Questionnaire about Presenteeism

		Strongly disagree	Somewhat disagree	Uncertain	Somewhat agree	Strongly agree
1.	Because of my health, the stresses of my job were much harder to handle.	( )	( )	( )	( )	( )
2.	Despite having stress, I was able to finish hard tasks in my work.	( )	( )	( )	( )	( )
3.	My work stresses distracted me from taking pleasure in my work.	( )	( )	( )	( )	( )
4.	I felt hopeless about finishing certain work tasks, due to my job stress.	( )	( )	( )	( )	( )
5.	At work, I was able to focus on achieving my goals despite my work stress.	( )	( )	( )	( )	( )
6.	Despite having my work stress, I felt energetic enough to complete all my work.	( )	( )	( )	( )	( )

Questionnaire related to innovative work behavior

INV1	Employees who come up with innovative projects in our hospital/Institute are encouraged to produce new projects even if they fail in the end.	1	2	3	4	5
INV 2	Creative opinions are supported in our hospital/Institute.	1	2	3	4	5

INV 3	Our hospital/Institute facilitates the increase of interdepartmental coordination activities for the development of innovation skills	1	2	3	4	5
INV 4	Our hospital/Institute prefers to adopt flexible organizational structures to develop innovation skills.	1	2	3	4	5
INV 5	Our hospital/Institute constantly enhances the business processes for the sake of better service and performance.	1	2	3	4	5
INV 6	Our hospital/Institute attaches importance to the training of the personnel on innovation and creativity methods.	1	2	3	4	5
INV 7	Additional rewards and incentives are given in our hospital/Institute to support the individuals who present successful and innovative projects.	1	2	3	4	5
INV 8	The employees of our hospital/Institute are not subject to punishment and sever criticism by upper management in case the new methods that they use fail.	1	2	3	4	5
INV 9	Team members are harmonious with each other in a collaborative environment rather than a competitive one in our hospital/Institute.	1	2	3	4	5
INV 10	Those who produce new and creative ideas in our hospital/Institute are awarded with premiums or promotion.	1	2	3	4	5
**competitors and technology**						
INV 11	Our hospital/Institute has launched more innovative products and services for the last five years than those of our competitors.	1	2	3	4	5
INV 12	The new products and services of our hospital/Institute mostly make us gain an advantage over our new competitors.	1	2	3	4	5
INV 13	Our hospital/Institute mostly benefits from the most advanced technology in the promotions of new products and services.	1	2	3	4	5
INV 14	The marketing methods we use for our products are revolutionary within the market compared to our competitors.	1	2	3	4	5
INV 15	Our hospital/Institute usually is the first one that brings a new product and service into the market.	1	2	3	4	5
INV 16	Our hospital/Institute management is willing to take risks to explore risky growth opportunities and not to miss them.	1	2	3	4	5
INV 17	The upper management of our hospital/Institute allocates enough time and resources for employees to produce new ideas.	1	2	3	4	5
**New services**						
INV 18	Our hospital/Institute has a lower success level of launching new products and services than our competitors.	1	2	3	4	5
INV 19	Our hospital/Institute's R&D or resources of product development are not enough to meet the need for developing new products and services.	1	2	3	4	5
INV20	In our hospital/Institute managers encourage their subordinates not to think much about the rules and procedures so that innovative ideas can be found.	1	2	3	4	5
INV21	The latest products and services of our hospital/Institute is a version of previous products and services.	1	2	3	4	5

Supervisor support

1	The supervisors strongly consider my goals and values	1	2	3	4	5
2	Help is available from supervisors when I have a problem	1	2	3	4	5
3	The supervisors really care about my wellbeing	1	2	3	4	5
4	The supervisors would forgive an honest mistake on my part	1	2	3	4	5
5	The supervisors are willing to help me when I need a special favor	1	2	3	4	5
6	If given the opportunity, the supervisors would take advantage of me	1	2	3	4	5
7	The supervisors show very little concern for me	1	2	3	4	5
8	The supervisors care about my opinions	1	2	3	4	5
